# Ca^2+^-Activated Cl^−^ Channels of the ClCa Family Express in the Cilia of a Subset of Rat Olfactory Sensory Neurons

**DOI:** 10.1371/journal.pone.0069295

**Published:** 2013-07-09

**Authors:** Carolina Gonzalez-Silva, Jorge Vera, María Rosa Bono, Christian González-Billault, Brooke Baxter, Anne Hansen, Robert Lopez, Emily A. Gibson, Diego Restrepo, Juan Bacigalupo

**Affiliations:** 1 Department of Biology, Faculty of Sciences, University of Chile, Las Palmeras, Santiago, Chile; 2 Institute for Cell Dynamics and Biotechnology, University of Chile, Las Palmeras, Santiago, Chile; 3 Department of Cell and Developmental Biology and Neuroscience Program, University of Colorado Anschutz Medical Center, Aurora, Colorado, United States of America; 4 Department of Bioengineering, University of Colorado Anschutz Medical Center, Aurora, Colorado, United States of America; Monell Chemical Senses Center, United States of America

## Abstract

The Ca^2+^-activated Cl^−^ channel is considered a key constituent of odor transduction. Odorant binding to a specific receptor in the cilia of olfactory sensory neurons (OSNs) triggers a cAMP cascade that mediates the opening of a cationic cyclic nucleotide-gated channel (CNG), allowing Ca^2+^ influx. Ca^2+^ ions activate Cl^−^ channels, generating a significant Cl^−^ efflux, with a large contribution to the receptor potential. The Anoctamin 2 channel (ANO2) is a major constituent of the Cl^−^ conductance, but its knock-out has no impairment of behavior and only slightly reduces field potential odorant responses of the olfactory epithelium. Likely, an additional Ca^2+^-activated Cl^−^ channel of unknown molecular identity is also involved. In addition to ANO2, we detected two members of the ClCa family of Ca^2+^-activated Cl^−^ channels in the rat olfactory epithelium, ClCa4l and ClCa2. These channels, also expressed in the central nervous system, may correspond to odorant transduction channels. Whole Sprague Dawley olfactory epithelium nested RT-PCR and single OSNs established that the mRNAs of both channels are expressed in OSNs. Real time RT-PCR and full length sequencing of amplified ClCa expressed in rat olfactory epithelium indicated that ClCa4l is the most abundant. Immunoblotting with an antibody recognizing both channels revealed immunoreactivity in the ciliary membrane. Immunochemistry of olfactory epithelium and OSNs confirmed their ciliary presence in a subset of olfactory sensory neurons. The evidence suggests that ClCa4l and ClCa2 might play a role in odorant transduction in rat olfactory cilia.

## Introduction

Olfactory transduction occurs in the cilia of olfactory sensory neurons (OSNs), where binding of odorant molecules to olfactory receptors triggers a cyclic AMP cascade. Mediated by heterotrimeric G-protein, G_olf_, the receptor stimulates adenylyl cyclase type III, rapidly increasing cAMP. This nucleotide directly gates cyclic nucleotide-gated nonselective cation channels, CNG, mediating a Ca^2+^ and Na^+^ influx into the ciliary lumen that contributes to the depolarizing current [1]. Ca^2+^ in turn gates Ca^2+^ activated Cl^−^ channels, allowing Cl^−^ efflux from the cilia. It is widely accepted that the Cl^−^ efflux makes up the largest fraction of the transduction current, reaching up to 85% in rat [2] and nearly 50% in amphibians [3].

The CNG channel has been extensively studied both molecularly as well as electrophysiologically [4]. It is made up of subunits (CNGA2, CNGB1b and CNGA4) that belong to the cyclic-nucleotide gated channel family, whose other well characterized member is found in vertebrate photoreceptors, where it plays a paramount role in visual transduction [5]. In contrast, much less is known about the Ca^2+^-activated Cl^−^ channels expressed in OSNs. Its biophysical features have only been partially characterized and a thorough pharmacological profile for this channel is unavailable. Moreover, its molecular identity continues to be a matter of debate. It is generally thought to be a small conductance channel, of 1 pS or less, with half-maximum Ca^2+^ concentration for activation (K_0.5_) in the low micromolar range, based on noise analysis studies of macroscopic currents recorded either from whole cilia [6], [7], or membrane patches excised from the dendritic knob, the terminal region of the OSN dendrite from where the olfactory cilia emanate, presumably populated by the same channels [8]. A member of the bestrophin Ca^2+^-activated Cl^−^ channel family, Best-2, was first proposed to correspond to the Ca^2+^-activated Cl^−^ transduction channel in mouse OSNs [9]. Nonetheless, further studies in knock-out mice lacking mBest-2 showed that the lack of this channel has no effect on odorant transduction [10], [11]. Recently, compelling evidence was provided supporting another Ca^2+^-activated Cl^−^ channel, belonging to the TMEM16 family, TMEM16B or ANO2 [12], [13] as the chemotransduction channel, based on RT-PCR, electrophysiological recordings in heterologous expression systems and immunohistochemical data [10], [14], [15]. Genetic disruption of this channel in mice largely affects the Ca^2+^-activated Cl^−^ current in dissociated mouse OSNs, supporting its involvement in odorant transduction [16]. However, the ANO2^−/−^ mice exhibited only a moderate reduction in field potential responses to odorants in the olfactory epithelium, as well as no behavioral impairment. It was concluded that the Ca^2+^-activated Cl^−^ conductance, presumably ANO2, was not required for odorant responses. Nevertheless, the contribution of an additional Ca^2+^-activated Cl^−^ conductance could explain the conflicting results of that study. Hence, the problem of the molecular identity of the Cl^−^ conductance that participates in olfactory transduction is not settled.

Here we report molecular biological and immunochemical studies performed in rat olfactory epithelia and in dissociated OSNs clearly showing the presence of two members of the ClCa Ca^2+^-activated Cl^−^ channel family in the cilia of a subset of OSNs (ClCa4l, GeneID: 499721 and ClCa2, GeneID: 362052). This evidence raises the possibility that these channels may participate in odorant transduction in the rat, in addition to ANO2. Perhaps the multiple species of Ca^2+^-activated Cl^−^ channels in olfactory cilia may reflect an evolutionary strategy that allows the olfactory system to overcome deleterious mutations in Ca^2+^-activated Cl^−^ channel genes that could affect this sensory modality, which is key for most vertebrates.

## Materials and Methods

### Animals

Sprague-Dawley male rats were obtained from the Pontificia Universidad Católica de Chile or the University of Colorado Anschutz Medical Campus animal facilities. All procedures conducted on the animals were in compliance with the University of Colorado Anschutz Medical Campus Institutional Animal Care and Use Committee. The IACUC specifically approved the procedures used in this study in animal protocol number B-39612(04)2D. In Chile, animal care and experimental procedures were approved by the Bio-Ethical Committee of the Faculty of Sciences, University of Chile, according to the ethical rules of the Biosafety Policy Manual of the National Fund for Scientific and Technological Development (FONDECYT).

### Design of Primers

The DNA sequences encoding for rat ClCa channels ClCa2 (GeneID: 362052, http://www.ncbi.nlm.nih.gov/gene/362052) and ClCa4l (GeneID: 499721, http://www.ncbi.nlm.nih.gov/gene/499721) have high identity (85%). For this reason we designed two pairs of primers capable of amplifying, using nested RT-PCR, DNA sequences encoding for both. The ClCa primers and the control CNGA2 (GeneID: 25411) primers are shown in Table S1. In addition, in order to determine the individual expression of ClCa2 and ClCa4l, we designed specific primers capable of amplifying through RT-PCR sequences for each DNA sequence (Table S2).

### Single Cell Nested RT-PCR

The rat olfactory epithelium was removed and mechanically dissociated; both olfactory sensory neurons (OSNs) and non-neuronal cells were morphologically identified under an Olympus IX70 microscope (100X phase contrast objective; Center Valley, PA). Patch-clamp pipettes were sterilized before use. After sealing the pipette against the cell, the OSN cytoplasm was harvested into the pipette under visual inspection, without losing the gigaseal. The pipette tip containing the cytoplasm was then broken into a sterile DNAse/RNAse-free PCR tube containing 16 µL of solution A (in mM, 1.25X PCR Buffer [200 Tris-HCl, 500 KCl] Invitrogen, 6.25 MgCl_2_ Invitrogen, 8 U RNAsin Promega, 2 DTT Invitrogen). 1U of DNAase (Promega; Madison WI) was added and the tube was incubated for 40 min at 37°C and subsequently for 10 min at 65°C. RT-reaction was performed after addition of 4 µL of RT mix to the samples. The RT mix contained 250 ng of random primers (Invitrogen, Carlsbad, CA), 1 mM dNTPs (Invitrogen), 40 U of RNAsin (Promega) and 200 U of reverse transcriptase (Superscript III, Invitrogen). cDNAs containing fractions were obtained after incubation of the mixture at 42°C for 30 min and then at 75°C for 5 min. As a negative control, reverse transcriptase was replaced by 1 µL of DEPC-water (Invitrogen). Following cDNA synthesis, the cDNA-containing solution was split in 3 aliquots. The first round of PCR was performed in a total volume of 30 µL with 1X PCR Buffer (Invitrogen), 0.2 mM dNTPs, 0.16 µM of each forward and reverse primers (Table S1 and S2) and 1U Platinum Taq polymerase (Invitrogen). Primers were designed for maximal specificity. The second round of amplification was performed in a total volume of 20 µL, with 10 µL of the first round PCR products, 1X PCR Buffer, 1.5 mM MgCl_2_ (Invitrogen), 0.2 mM dNTPs (Invitrogen), 0.16 µM of each forward and reverse nested primers (Table S1) and 1U Platinum Taq polymerase. The PCR protocol consisted of 40 cycles with 30 s of denaturation for the first and the second round, 45 s of annealing for the first and 30 s for the second round, followed by 1 min extension for the first round and 30 s for the second. PCR products for ClCa and CNGA2 were examined in ethidium bromide stained agarose gels after electrophoresis to verify expected sizes. The ClCa band were isolated using the QIAquick Gel Extraction Kit (QIAGEN, Venlo, Netherlands) according to manufacturer’s instructions, sequenced at Macrogen (http://dna.macrogen.com/eng/) and aligned against a database using Geneious v5.3 (www.geneious.com) and ClustalW (www.ebi.ac.uk/Tools/msa/clustalw2).

### Olfactory Epithelium (OE) and Brain Nested RT-PCR

OE and brain RNA were isolated using the RNeasy Mini Kit (QIAGEN). In order to avoid genomic DNA contamination, the total RNA extract was further treated with RNAase-free DNAase I (QIAGEN). Nested PCR reactions were performed using the same methodology described in the previous section, with specific primers for ClCa2 and ClCa4l (Table S2). Relative abundance of ClCa2 and ClCa4l transcripts in rat OE was estimated using a nested PCR protocol with 30 cycles for the first and 35 cycles for the second round of amplification. Small aliquots were collected in the second round of amplification at 5, 10, 15, 20, 25, 30 and 35 cycles and further analyzed in ethidium-bromide containing agarose gels.

The ClCa species was analyzed by PCR reaction using seven different overlapping primers (capable of amplifying both ClCa2 and ClCa4l, Table S3) spanning the whole ClCa mRNA. PCR products were purified, sequenced and aligned against ClCa2 (GeneID: 362052) and ClCa4l (GeneID: 499721) using “Genious Basic 3.7.1″ software Geneious v5.3 (www.geneious.com) or ClustalW (www.ebi.ac.uk/Tools/msa/clustalw2).

### Western Blot

OSN-derived cilia protein extracts were performed as previously described [17]. Protein concentration was determined by a colorimetric method [18]. 50 mg of protein extracts were loaded and run in 8% polyacrylamide gels at 120 V. The proteins were then transferred to nitrocellulose membranes in 120 mM Glycine, 125 mM Tris, 0.1% SDS and 20% methanol, at 150 mA for 1.5 hour. Then filters were blocked in 5% non-fat dry milk in PBS containing 0.1% Tween-20 for 2 hours at room temperature and incubated overnight with primary antibodies diluted in 5% non-fat dry milk in PBS containing 0.1% Tween-20 at 4°C. Membranes were incubated with the following antibodies: anti-ClCa 1∶200 and anti-β-actin 1∶250 (Sigma, catalogue number A3853; St Louis, MO). Secondary antibody was a goat anti-mouse IgG-HRP (Thermo Scientific, catalogue number 31430) used at a dilution of 1∶10000 in blocking solution. Membranes were washed three times with PBS containing 0.1% Tween-20 for 15 minutes and then labeling was visualized with ECL reagent (Thermo Scientific, catalogue number 32106). All the Western blot data are representative of at least three independent experiments. OE and Brain (positive control) samples were prepared using the “ProteoJET Mammalian Cell Lysis Reagent” (Fermentas, Vilnius, Lithuania) according to manufacturer’s instructions.

### Monoclonal Antibody Preparation

In order to generate the anti-ClCa antibody, we used a 17 amino acid peptide (SKSEYLMPKRESYDKAK) [19]. The peptide was coupled to CCH (Blue carrier, Biosonda, Santiago, Chile) or Ovalbumin following the manufacturer’s instructions. Mice were immunized intraperitoneally with 200 µL of peptide-CCH emulsified in Complete Freund's adjuvant and they were boosted weekly with 200 µL of peptide-CCH emulsified in incomplete Freund's Adjuvant until the titer of the serum was higher than 1/1000. Hybridomas were prepared by fusing NSO/2 mice myeloma cell line with splenocytes from immunized mice by a modified protocol from Kohler and Milstein [20]. Screening of the hybridomas was performed by ELISA, testing them with the peptide-CCH and peptide-OVA as a negative control. Selected hybridomas were further analyzed by Western blot and were sub-cloned by limiting dilution.

### Immunohistochemistry

Wild type three week old (Sprague-Dawley) rats were anesthetized with nembutal (100 mg/kg), perfused transcardially with 0.9% saline followed by 4% paraformaldehyde, pH 7.2. The olfactory organs were dissected and postfixed in the same fixative for 5 hours. Cryoprotection was carried out in 20% sucrose overnight. The tissue was embedded in Tissue Tek OCT (Sakura Finetek, Torrance, CA). Cryosections (12–14 µm) were mounted on Superfrost Plus slides (VWR, West Chester, PA) and frozen at −80°C until further use. Standard immunohistochemical procedures were used. Briefly, cells were washed in 0.1 M phosphate buffered saline (PBS), blocked in blocking solution containing 1% BSA, 3% normal serum, and 0.3% Triton X-100 in PBS for 2 hours, and then incubated in the primary antisera overnight (anti-ClCa antibody, 1∶100; anti-CNGA2 goat IgG antibody, Santa Cruz sc-13700, 1∶100; monoclonal acetylated tubulin, Sigma T7451, 1∶1000, rabbit IgG against ANO2, 1∶1000 [16]). After 3 washes, 20 min each, the cells were incubated in the appropriate secondary antibodies (Alexa 488, Alexa 568, 1∶400; Invitrogen, Carlsbad, CA) for 2 hours at room temperature. After incubation, sections were washed 3 times 20 min and coverslipped with Fluormount-G (Fisher Biotech, Birmingham, AL). Control slides were treated either without the primary antibody or with normal rabbit serum replacing the primary antiserum. Control sections showed no labeling.

For immunocytochemistry with isolated cells, Sprague-Dawley rats of 3 weeks of age were euthanized by CO_2_ inhalation followed by cervical dislocation. Cell isolation was as in Rawson et al. [21]. Briefly, olfactory epithelium was removed from the animal, cut into small pieces and placed in Ca-Mg-free mouse Ringer with 10–30 U/ml of papain and 2 mM cysteine for 15 min at room temperature. The cells were detached by gentle pipetting and filtered through nylon mesh to remove connective tissue. The cells were then placed on Superfrost Plus slides (VWR, West Chester, PA) slides coated with Cell-Tak (BD Bioscence, catalogue number: 354242) and 1 mg/ml concanavalin A (Sigma, 11028-71-0) and they were allowed to settle for 30 min. At this point cells were fixed with 4% paraformaldehyde in PBS pH 7.2 for 20 min.

Standard immunohistochemical procedures were used. Briefly, cryosections were washed in 0.1 M phosphate buffered saline (PBS), blocked in blocking solution containing 0.3% BSA, 0.3% normal serum, and 0.05% Triton X-100 in PBS for 30 min, and then incubated in the primary antisera overnight (anti-ClCa antibody, 1∶100; anti-CNGA2, Santa Cruz sc-13700, Lot J1305; 1∶500; rabbit polyclonal anti-ANO2 IgG antibody generated by Billig and co-workers; 1/1000 [16]). We tested anti-ClCa antibody concentrations from 1∶100 to 1∶500 dilutions, with similar results regardless of the concentration. After 3 washes, 10 min each, the sections were incubated in the appropriate secondary antibodies (Alexa 488, Alexa 555, 1∶400; Invitrogen) for 2 hours at room temperature. After incubation, sections were washed 3 times, 5 min each, and coverslipped with Fluormount-G (Fisher Biotech). Control slides were treated without the primary antibody. Control slides showed no labeling.

Tissue sections and isolated cells were viewed under a fluorescence microscope or a confocal laser microscope (Olympus). Unless otherwise stated the images shown are the result of a z stack encompassing the entire object shown (OSNs or olfactory epithelium). Figures were created in Adobe Photoshop, a Version CS2 (Adobe Systems Inc., San Jose, CA). The figures for the isolated cells were deconvolved in MATLAB using the Richardson-Lucy deconvolution method using psf determined using 200 nm beads [22]. Results of immunostaining presented here were obtained from six rats and one mouse.

## Results

Two ClCa channels (ClCa2 and ClCa4l) are expressed in the rat nervous system [19], [23]. This led us to determine whether any of these members of the ClCa family are present in the olfactory epithelium and more specifically in the OSNs.

**Figure 1:**
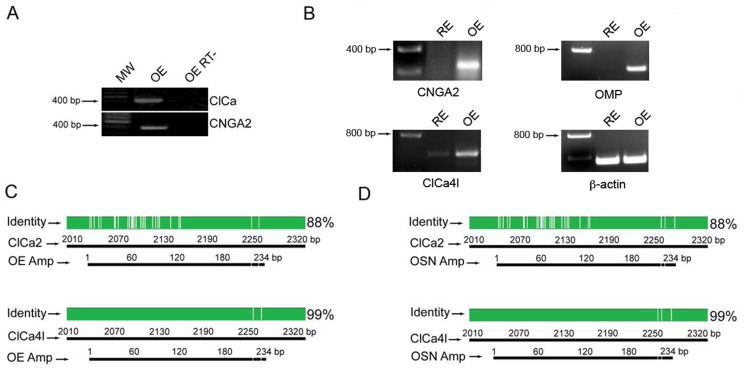
ClCa channel is expressed in rat olfactory sensory neurons. A. RT-PCR of rat olfactory epithelium (OE), using generic primers for ClCa4l and ClCa2 (ClCa). Positive control: CNGA2. OE RT-: Reverse transcriptase-free samples. MW: molecular weight marker (GeneRuler 100 bp plus DNA Ladder, Fermentas). B. RT-PCR from mRNA isolated from the olfactory (OE) and respiratory epithelia (RE). C. Alignment of ClCa amplicon of olfactory epithelium with ClCa2 and ClCa4l. Green denotes identity and white denotes difference in nucleotide sequence. D. Alignment of ClCa amplicon from an olfactory sensory neuron with ClCa2 and ClCa4l.

Expression of mRNA encoding for the ClCa channels ClCa2 and ClCa4l was probed in the olfactory epithelium by means of RT-PCR using generic primers for the ClCa2/4l transcripts (ClCa primers in Table S1). As shown in Fig. 1A, mRNA encoding for ClCa is expressed in the olfactory epithelium, and Fig. 1B shows that ClCa4l mRNA is expressed at low levels in respiratory epithelium compared to the olfactory epithelium. The principal subunit of the CNG channel CNGA2 and the olfactory marker protein (OMP) used as control, were also detected in the olfactory epithelium but not in the respiratory epithelium and β-actin was expressed in both epithelia.

The 400 bp amplicon obtained from the olfactory epithelium was sequenced and aligned with the sequences of ClCa channels ClCa2 and ClCa4l, and not with other ClCa channel sequences. The amplicon had 88% identity with ClCa2 and 99% with ClCa4l (Fig. 1C). The same procedure was also followed with the amplicon derived from the single-cell nested RT-PCR of OSNs with identical result (Fig. 1D), confirming that the amplicons obtained from both sources were the same. This evidence indicates that the transcript for the ClCa4l protein was expressed in olfactory sensory neurons.

**Figure 2:**
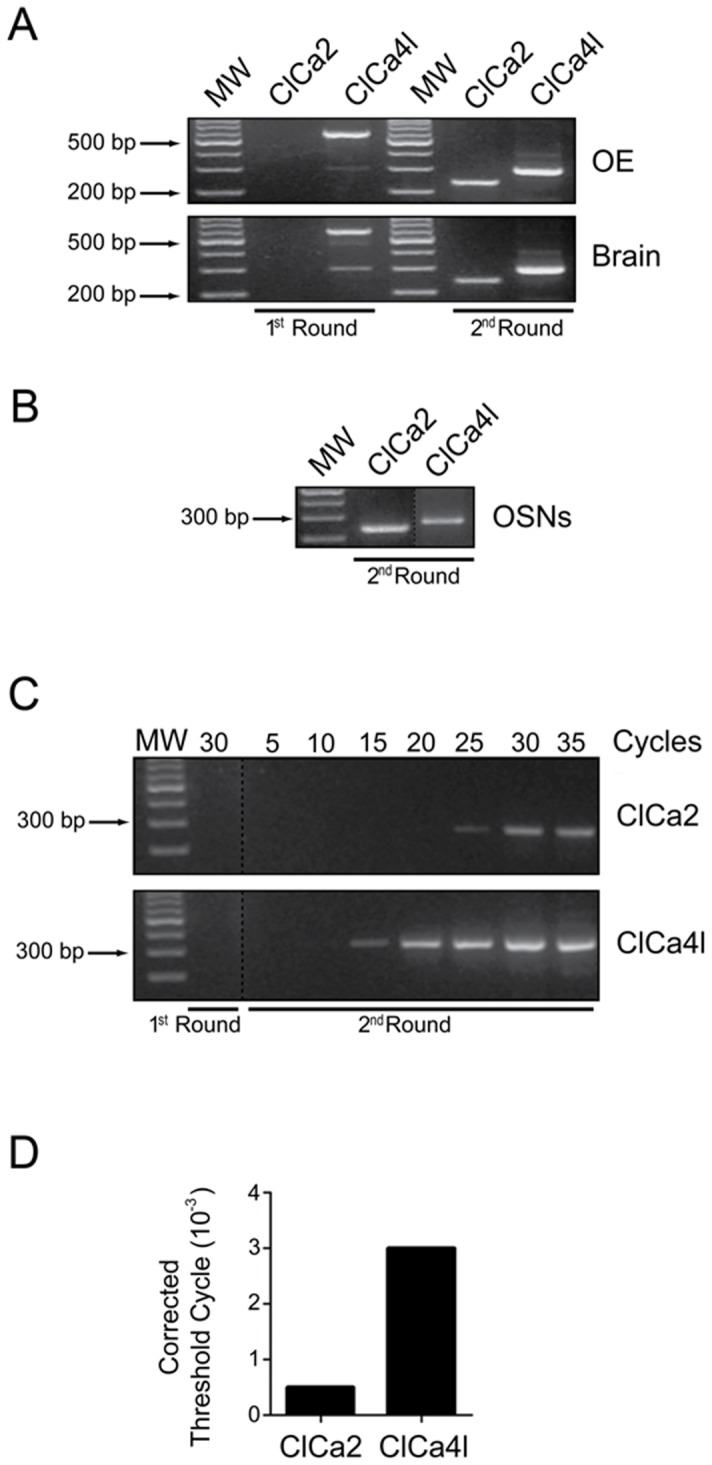
ClCa2 and ClCa4l are expressed in rat olfactory epithelium and olfactory sensory neuron. A. ClCa2 and ClCa4l mRNA expression in rat olfactory epithelium after the first (1^st^) and second (2^nd^) round of nested RT-PCR. We used a brain as positive control. B. Expression of both mRNAs in an isolated olfactory sensory neuron. RT-: Reverse transcriptase-free samples. MW: molecular weight marker (GeneRuler 100 bp plus DNA Ladder, Fermentas). C. Using the respective primers for each of the channels we conducted a first round of PCR (30 cycles) and a second round (35 cycles), from which we obtained samples in cycles 5, 10, 15, 20, 25, 30 and 35. D. RT q-PCR was used for determining the abundance of ClCa4l and ClCa2, using a single round with 35 cycles. The threshold cycle values were corrected with the rat housekeeping YWHAZ.

To further examine whether there was expression of both channels, ClCa4l and ClCa2, in the olfactory epithelium, we designed specific primers for each of them (Table S2) and ran nested RT-PCR experiments for olfactory epithelium and OSNs. We found a clear expression of mRNA encoding for both proteins in the two preparations, indicating that both ClCa4l and ClCa2 are expressed in the epithelium (Fig. 2A) and specifically in OSNs (Fig. 2B). In addition, we performed nested RT-PCR to assay the relative levels of expression of mRNA encoding for these channels in OE. The second round of nested RT-PCR revealed that ClCa2 bands only became evident in the 25^th^ cycle, while ClCa4l were already visible in the 15^th^ cycle, an indication that the latter mRNA was more abundant than the former (Fig. 2C). In order to add additional evidence to the identification of the highest expression ClCa channel species in the olfactory epithelium, we carried out real time RT-PCR experiments (Fig. 2D). The real time RT-PCR plot reveals a substantial difference in cycle threshold, indicating that ClCa4l mRNA is expressed more abundantly than ClCa2 mRNA in rat olfactory epithelium.

**Figure 3:**
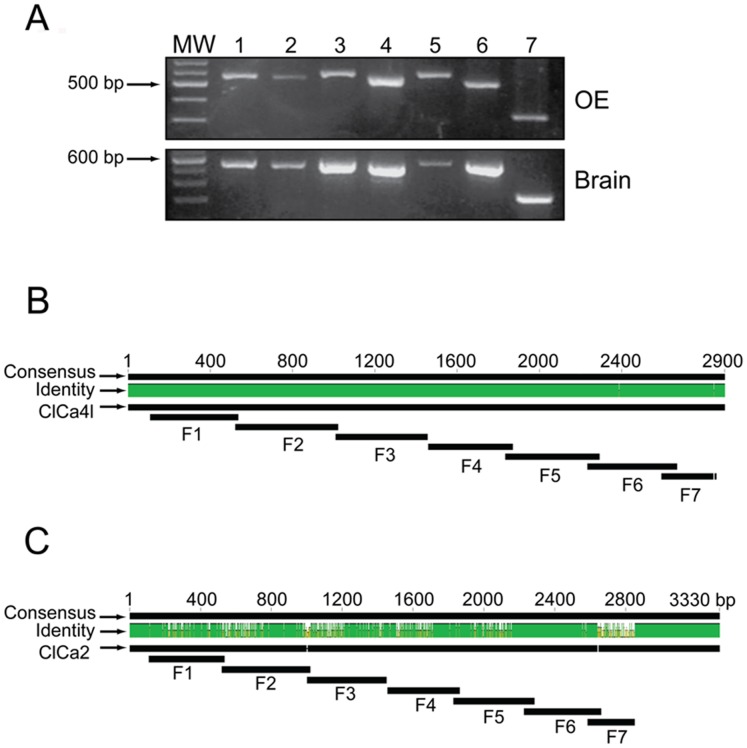
Complete sequence of ClCa channels in rat OE. A. Amplicons of seven fragments comprising the entire cDNA sequence of the ClCa2 and ClCa4l channels of rat olfactory epithelium and brain (positive control). B. The black boxes correspond to the nucleotide alignment of the seven cDNA fragments (F1 to F7) with the ClCa4l sequence. Green denotes identity and white denotes difference in nucleotide sequence. C. Nucleotide alignment of the seven cDNA fragments (F1 to F7) with the ClCa2 sequence.

In order to determine whether the most abundant cDNA of ClCa corresponded to the published ClCa4l protein and not to a variant of it, we prepared seven pairs of primers (Table S3) that altogether covered the whole sequence of ClCa2 and ClCa4l. The seven amplicons (Fig. 3A) were aligned with the ClCa sequences. Identity with ClCa4l was nearly 100% with only a single, T2387C, transition (Fig. 3B, Fig. S1) whereas with ClCa2 the identity was lower in all segments (Fig. 3C), indicating that the most abundant expressed mRNA sequence corresponds exactly to the ClCa4l channel protein.

**Figure 4:**
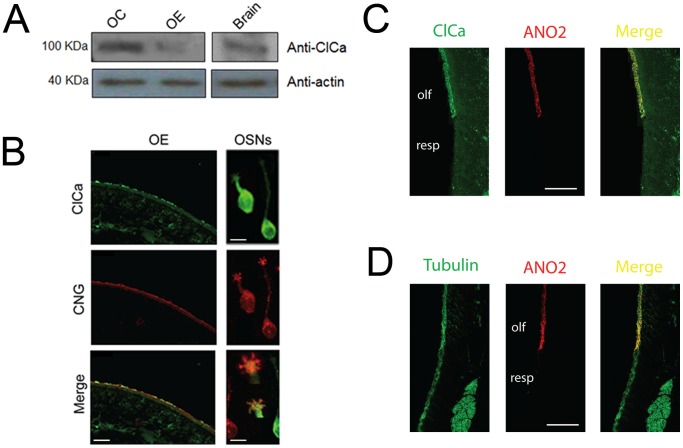
Western blot and immunohistochemical localization of ClCa to the olfactory cilia. A. Western blots of an enriched ciliary membrane preparation (OC), olfactory epithelium membranes (OE) and brain (positive control) samples using the anti-ClCa and the anti-actin antibodies. B. Immunolabeling of the olfactory epithelium (left panels) and of dissociated olfactory neurons (right panels) using specific anti-ClCa (green) and anti-CNGA2 (red) antibodies. Please note that in the dissociated cells the antibody immunoreactivity was found in the cilia, but there was also immunoreactivity in the cell body likely due to leakage of the ciliary proteins to the cell body after dissociation. Calibration bars in B: left, 10 µm; right top, center, 10 µm; right bottom, 2 µm. C and D. Immunolabeling of the olfactory and respiratory epithelia by anti-ClCa, anti-ANO2 known to label the olfactory cilia [16] and anti-acetylated tubulin that labels the cilia layer in both the olfactory and respiratory epithelia. Bars are 50 µm. C. The ciliary layer of the olfactory epithelium is labeled by both anti-ClCa (green) and anti-ANO2 (red). D. Both the respiratory and olfactory ciliary layers are labeled by anti-acetylated tubulin (green). The olfactory epithelium is identified by labeling with anti-ANO2 (red).

At this stage, it was important to unravel whether the protein encoded by the ClCa mRNA under study was expressed in OSNs and, more specifically, in the chemosensory cilia. We generated a monoclonal antibody against a 20 amino acid peptide previously used to detect ClCa2 in rat salivary gland [19]. Since ClCa4l and ClCa2 sequences display high similarity, this antibody detects both proteins. Western blots of membranes enriched in ciliary membranes exhibited immunoreactivity for a 100 kD protein, corresponding to the molecular weight of ClCa. The membrane preparation obtained from the entire epithelium and that from brain (positive control) showed a weaker reactivity than the ciliary fraction, as expected because ClCa was more diluted than in the ciliary fraction (Fig. 4A). This result is indicative that at least one of the channels is present in the cilia.

As an independent approach to investigate the localization of ClCa in the olfactory epithelium, we performed immunohistochemistry with the anti-ClCa antibody on 12 µm cryosections of rat olfactory epithelium. As shown in Fig. 4B, the antibody labeled patches in the ciliary layer, indicating expression in a subset of the cells contributing apical endings in this layer. An anti-CNGA2 antibody used as a positive control labeled the ciliary layer uniformly. In order to determine whether the protein was expressed in the cilia of OSNs, we also performed immunocytochemistry in dissociated OSNs, where the anti-ClCa antibody immunoreacted against the chemosensory cilia in 30% of the isolated OSNs (n = 98). In contrast, CNGA2 antibody labeled the cilia in every OSN (Figs. 4B and S3). The morphology of OSNs labeled and not labeled with anti-ClCa did not differ. Importantly, survey of the slides by difference interference contrast revealed that all cells with OSN morphology were also labeled with CNGA2 antibody (and ∼1/3^rd^ labeled anti-ClCa antibody). In addition, a large number of cells with morphology different from OSNs did not label with either antibody (Fig. S3, E–G) and while the ciliary layer of the olfactory epithelium labeled with antibodies against ANO2, CNGA2 (not shown) and ClCa none of these antibodies labeled the ciliary layer in the respiratory epithelium that was labeled by an antibody against acetylated tubulin (Fig. S4). Finally, when olfactory epithelium was stained with both antiANO2 and anti-ClCa and was viewed at high magnification in the confocal microscope it was found that although there were areas of the cilia layer where there was overlapping staining there also were some areas where the staining was not overlapping (see arrows in Fig. S4) indicating that these antibodies recognize two different Ca^2+^-activated Cl^−^ channels expressed non-uniformly in cilia. Parallel experiments showed expression of ClCa in cilia of mouse OSNs (not shown). These results show that ClCa is found in the chemosensory cilia of a subset of rat OSNs.

It has been shown that mouse olfactory epithelium expresses the Ca^2+^-activated Cl^−^ channel ANO2 (14). We examined, by means of nested RT-PCR, whether a transcript encoding for this channel was also expressed in rat. We confirmed the presence of a transcript for ANO2 and ANO1 in rat olfactory epithelium (Fig. S2A, Table S4). In addition, by immunohistochemistry we detected the presence of ANO2 in the cilia of OSNs (Fig. S2B).

## Discussion

The key role for Ca^2+^-activated Cl^−^ channels in olfactory transduction has been widely documented in vertebrate OSNs, from amphibians to mammals. In the present work we demonstrate that mRNA transcripts for two members of the ClCa family, ClCa4l and ClCa2, are expressed in rat OSNs. Furthermore, we show that immunohistochemistry indicates that ClCa channel proteins are present in the chemosensory cilia of a subset of OSNs, opening the possibility that they contribute to the odorant transduction Cl^−^ conductance.

Among the ClCa channel family members that have been identified in the rat central nervous system [19], [23], mRNAs for ClCa1/2 and ClCa4l have been found in the olfactory epithelium and olfactory nerve in the mouse embryo by *in situ* hybridization, and have been postulated to be expressed in olfactory ensheathing cells [24]. Here we demonstrate that two members of the ClCa Ca^2+^-activated Cl^−^ channel family, ClCa4l and ClCa2, are expressed in rat olfactory epithelium, as indicated by RT-PCR experiments. Moreover, the single-cell RT-PCR studies presented here indicate that both genes are expressed specifically in the OSNs and that ClCa4l mRNA is more abundant than ClCa2, an observation that was confirmed by real time RT-PCR. Moreover, we also show, by immunohistochemistry of rat olfactory epithelium cryosections as well as dissociated OSNs, and immunoblotting of a membrane fraction enriched in olfactory cilia [17], that the products of one or both of these genes, namely ClCa4l and ClCa2, are localized to the cilia membrane, where they are expected to be if they are involved in chemotransduction. A protein Blast of the rat proteome shows that the peptide used to generate the ClCa antibody with 100% identity with the ClCa4l protein sequence has below 4 amino acid identity with ANO1 (3 amino acids) and ANO2 (2 amino acids). Thus the immunohistochemistry with ClCa antibody would not detect ANO2 or ANO1 [25]. Importantly data showing that there are ciliary layer areas labeled by the ClCa antibody but not by the ANO2 antibody (Fig. S4) corroborate that the ANO2 and ClCa antibodies bind to different proteins. Finally, our finding that the only mRNA transcript for the ClCa family found in olfactory tissue is 100% identical with ClCa4l suggests that this is the ClCa channel expressed in OSNs. Although this represents strong evidence, it is insufficient to demonstrate ClCa channels are effectively taking part in transduction. Future functional experiments and loss of function studies are necessary for establishing which of all candidates for the transduction channel is functionally relevant.

In recent years two proteins belonging to the superfamily of Ca^2+^-activated Cl^−^ channels have been proposed to correspond to the olfactory Ca^2+^-activated Cl^−^ channels, Bestrophin-2 [9] and TMEM16B, also known as ANO2 or Anoctamin 2 [14]. Bestrophin was found in the ciliary layer of mice olfactory epithelium. Electrophysiological studies suggested a small conductance channel (0.26 pS), with a K_0.5_ for Ca^2+^ (0.4 µM) an order of magnitude smaller than the native channel (5 µM) [6], and an ion selectivity sequence and pharmacological profile resembling the olfactory channel, although no specific blockers for Ca^2+^-activated Cl^−^ channels are available to date. However, more recent reports indicated that a knock-out mouse lacking this channel exhibited a normal sense of olfaction [26] as well as normal electrophysiological odorant responses [11]. This evidence was taken to show that Best-2 does not make a relevant contribution to olfactory transduction. In a more recent study, Stephan et al. [14] provided evidence for the ANO2 channel as a novel candidate for the olfactory Ca^2+^-activated Cl^−^ channel. These authors identified ANO2 from a proteomic analysis of a mouse olfactory cilia preparation. This channel was heterologously expressed in HEK-293 cells system, from where its unitary conductance, derived by noise analysis, was estimated to be 0.8 pS and its K_0.5_ for Ca^2+^1.83 µM, consistent with previous noise analysis measurements performed on excised dendritic knob membrane patches (γ = 1.27 pS, K_0.5_ = 1.83 µM) (8) and on excised cilia (γ = 1.6 pS) [27]. The selectivity sequence for anions also matched that of the Cl^−^ transduction channel. In addition, *in situ* hybridization studies determined the presence of ANO2 in the ciliary layer of the olfactory epithelium [14]. Indeed, here we show that ANO2 is expressed in the cilia of OSNs (Fig. S2). This finding has recently received strong support from studies on mice in which the *ANO2* gene was genetically disrupted [16]. This study showed that the Ca^2+^-activated component was virtually absent in whole cell current recordings from the tested individual ANO2^−/−^ olfactory neurons. Nevertheless, those animals exhibited normal EOGs as well as unimpaired olfactory behavior. Altogether, the evidence is controversial regarding the extent of ANO2 involvement in odorant transduction. The lack of an effect of knockout of Ca^2+^-activated Cl^−^ channels on olfactory behavior and its modest alteration of EOGs could be due to the lack of a role of Ca^2+^-activated Cl^−^ channels in olfactory transduction *in situ*. An alternative explanation for these conflicting results is that the epithelial odorant responses remaining after knocking ANO2 may be mediated by a subpopulation of OSNs that express the ClCas, which may have been missed in the single cell studies due to the relatively small sample of cells tested. This latter possibility is consistent with the observations of patches of ClCa expression observed in our immunohistochemistry experiments. Future loss of function studies with multiple Ca^2+^-activated Cl^−^ channels may be necessary to definitively settle this issue. In addition, the lack of single-channel data from this and the other two Ca^2+^-activated Cl^−^ channels (ClCa and Best-2) with the possibility of corresponding to the olfactory Cl^−^ channel has been an important limiting factor for its identification.

It may seem intriguing that the cilia appear to express multiple Ca^2+^-activated Cl^−^ channel species, perhaps as many as three, with one channel ClCa expressed in a subset of OSNs. In addition, the cilia layer of the olfactory epithelium expresses another Ca^2+^-activated channel, the transient receptor potential channel M5 (TRPM5), suggesting its presence in olfactory cilia [28]. This suggests redundancy of Ca^2+^-activated channels in OSNs. Importantly, the redundancy of protein expression is not at all of rare occurrence. It has been shown that olfactory cilia express at least three types of Ca^2+^-activated K^+^ channels, of small, intermediate and large conductances [29] and that olfactory cilia express two types of Ca^2+^ transporters, a Na^+^/Ca^2+^ exchanger [30] and a Ca^2+^-ATPase [31]–[33] and two types of Cl^−^ transporters, NNKCC1 and Ca^2+^/HCO_3_^−^ [34]. Our experiments demonstrate expression of both ANO2 and ClCa in the cilia of olfactory sensory neurons. This finding of two ciliary Ca^2+^-activated Cl^−^ channels casts a shadow on whether single gene knockout experiments [16] will provide information on the role of these different channels in olfactory transduction. In the case of the olfactory Ca^2+^-activated Cl^−^ channel, it seems tempting to speculate that a redundancy of Ca^2+^-activated chloride channel types may be an evolutionary mechanism by which animals, for which olfaction is a most critical sense, may afford the loss of one Ca^2+^-activated Cl^−^ channel type as a result of mutations.

## Supporting Information

Figure S1 **Alignment for each PCR fragment amplified with the primers shown in Table S3 with the ClCa4l sequence (GeneID: 499721, see also** **Fig. 3** **).**
(TIF)

Figure S2 **PCR and immunohistochemical studies consistent with the presence of ANO2 in the cilia of olfactory sensory neurons.** A. PCR fragments amplified with primers shown in Table S4. B. Immunohistochemical demonstration of the presence of ANO2 (green) and CNGA2 (red) in the cilia of an isolated olfactory sensory neuron. This is representative of images obtained from 22 OSNs.
(TIF)

Figure S3 **Four examples of immunolabeling of dissociated olfactory neurons (A–D) and lack of immunolabeling of cells with morphology different from OSNs (E–H).** OSNs in A and B do not show immunolabeling to ClCa in the cilia while those in C and D do immunolabel for ciliary ClCa. A–E and G were co-labeled with anti-ClCa (green) and anti-CNGA2 (red) antibodies. E and G are DIC corresponding to F and H. Bars are 5 µm.
(TIF)

Figure S4 **High magnification image of the ciliary layer of the olfactory epithelium with anti-ClCa (green), anti-ANO2 (red).** While the cilia layer is labeled by both antibodies with significant overlap there are areas of the ciliary layer where these two antibodies do not overlap (arrows). Bar is 10 µm. This was obtained as a single image with a 60x oil objective.
(TIF)

Table S1 **Information for nested primers for ClCa and CNGA2 (** **Fig. 1** **).** The table includes the sequence and specification of each nested primer used to amplify mRNA of ClCa and CNGA2 to obtain the PCR products shown in Fig. 1. Forward (F) and reverse (R) primers for the first (F1/R1) and second (F2/R2) rounds of amplification are included.
(JPG)

Table S2 **Information on specific nested primers for ClCa2 and ClCa4l (** **Fig. 2** **).** The table shows the sequence of the specific ClCa2 and ClCa4l nested primers.
(TIF)

Table S3 **Primer information for PCR used to determine the full sequence of the olfactory ClCa transcript.** The table shows information on the seven pairs of primers used to amplify the entire sequence of the olfactory epithelium ClCa mRNA. The position refers to the sequence of ClCa4l (GeneID: 499721). These fragments and the alignment of the sequence with ClCa2 (GeneID: 362052) and ClCa4l are shown in a graphic form in Fig. 3. In addition, the sequences of each fragment with ClCa4l are shown in Fig. S1. Because of their high sequence similarity these primers would be able to amplify PCR products from both ClCa4l and ClCa2.
(TIF)

Table S4 **Information on specific primers for ANO1 and ANO2 (Fig. S2).** The table shows the sequence of the specific ANO1 (GeneID: 309135) and ANO2 (GeneID: 243634) primers.
(TIF)
