# Supplementary figures and images for: Ca2+-Activated Cl− Channels of the ClCa Family Express in the Cilia of a Subset of Rat Olfactory Sensory Neurons
Source: PLoS One. 2013 Jul 9;8(7):e69295. doi: 10.1371/journal.pone.0069295 (PMC3706372; doi:10.1371/journal.pone.0069295)

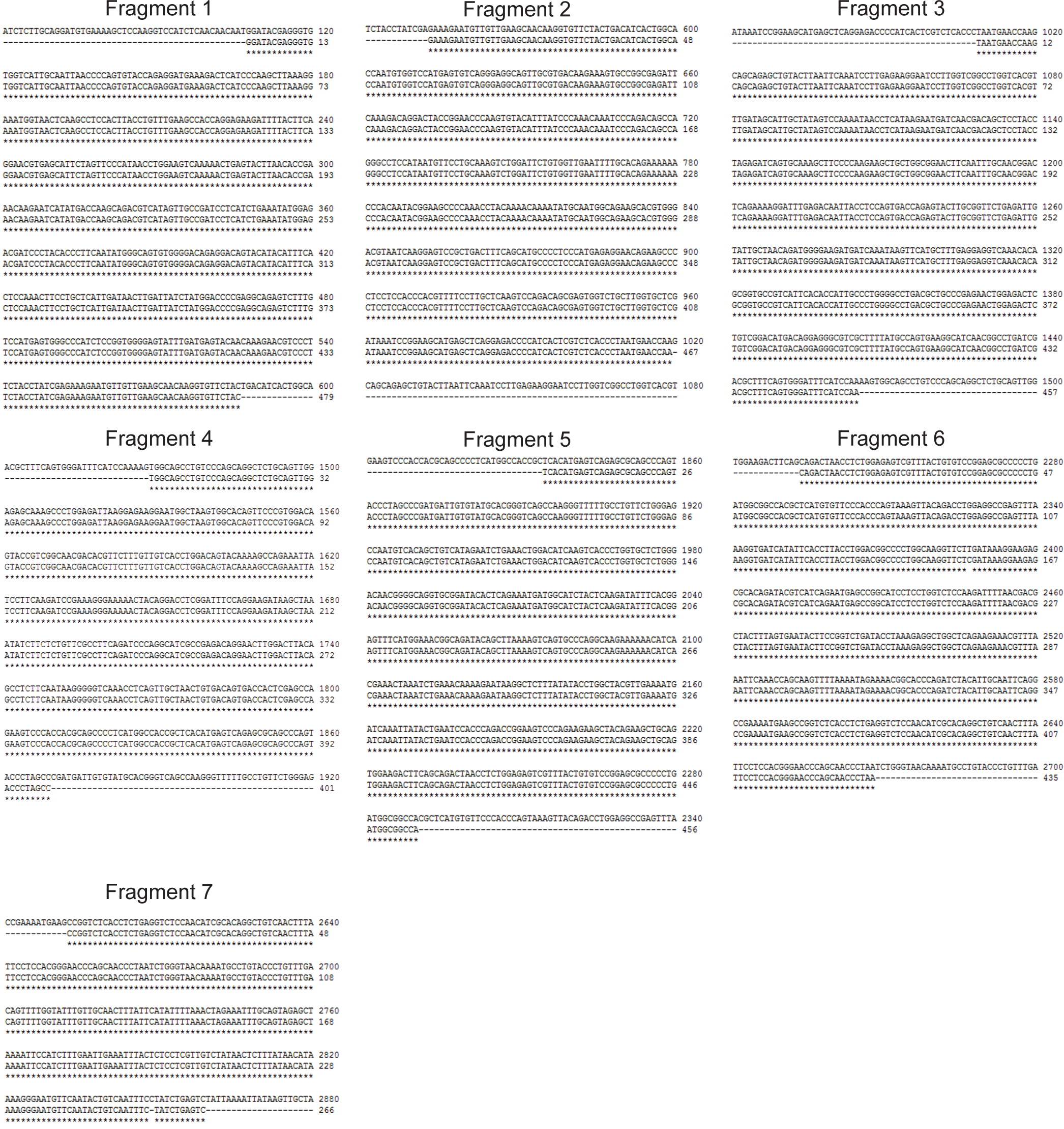

Supplement: Figure S1 — Alignment for each PCR fragment amplified with the primers shown in Table S3 with the ClCa4l sequence (GeneID: 499721, see also Fig. 3 ). (TIF) [file pone.0069295.s001.tif]

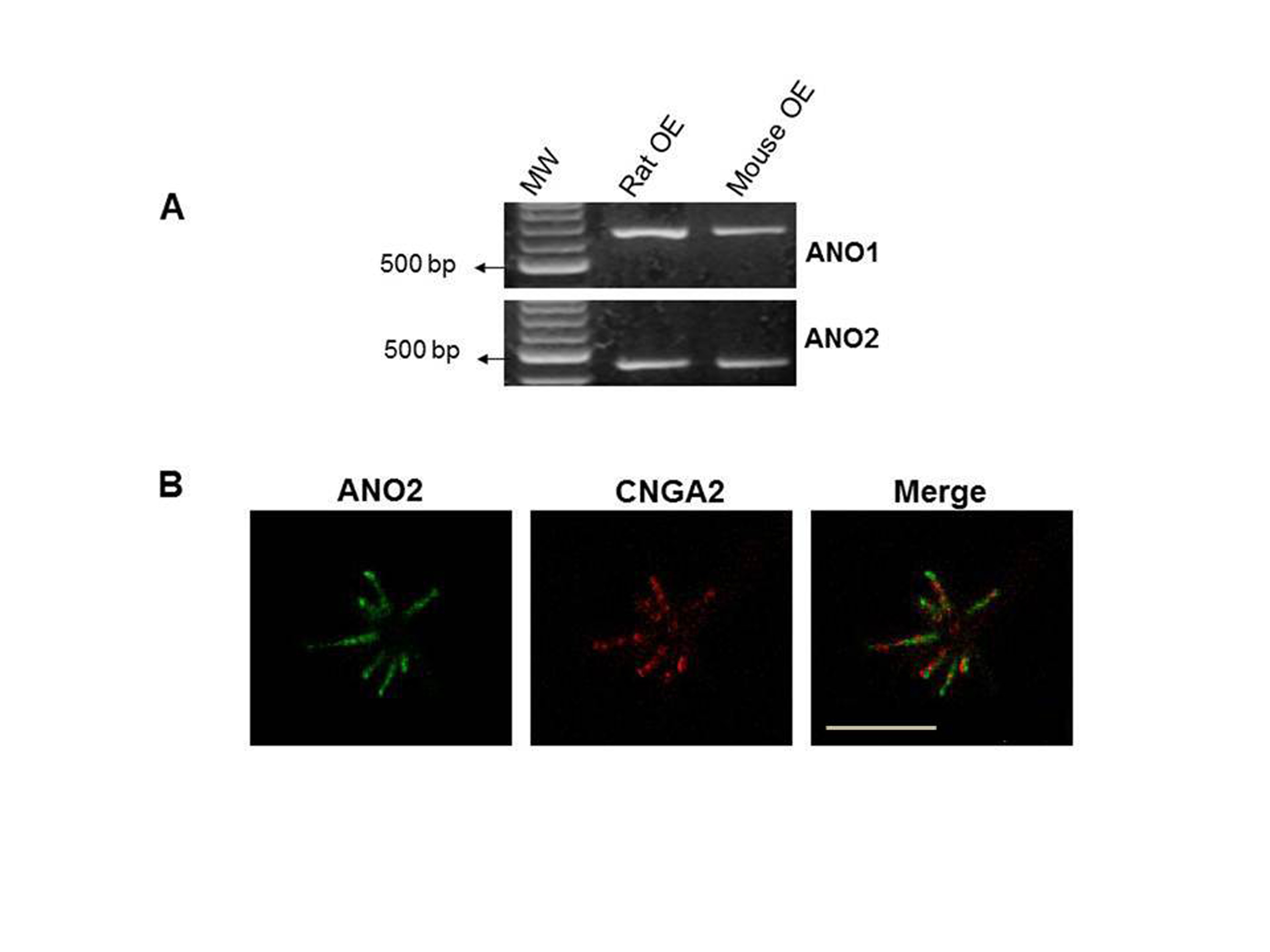

Supplement: Figure S2 — PCR and immunohistochemical studies consistent with the presence of ANO2 in the cilia of olfactory sensory neurons. A. PCR fragments amplified with primers shown in Table S4. B. Immunohistochemical demonstration of the presence of ANO2 (green) and CNGA2 (red) in the cilia of an isolated olfactory sensory neuron. This is representative of images obtained from 22 OSNs. (TIF) [file pone.0069295.s002.tif]

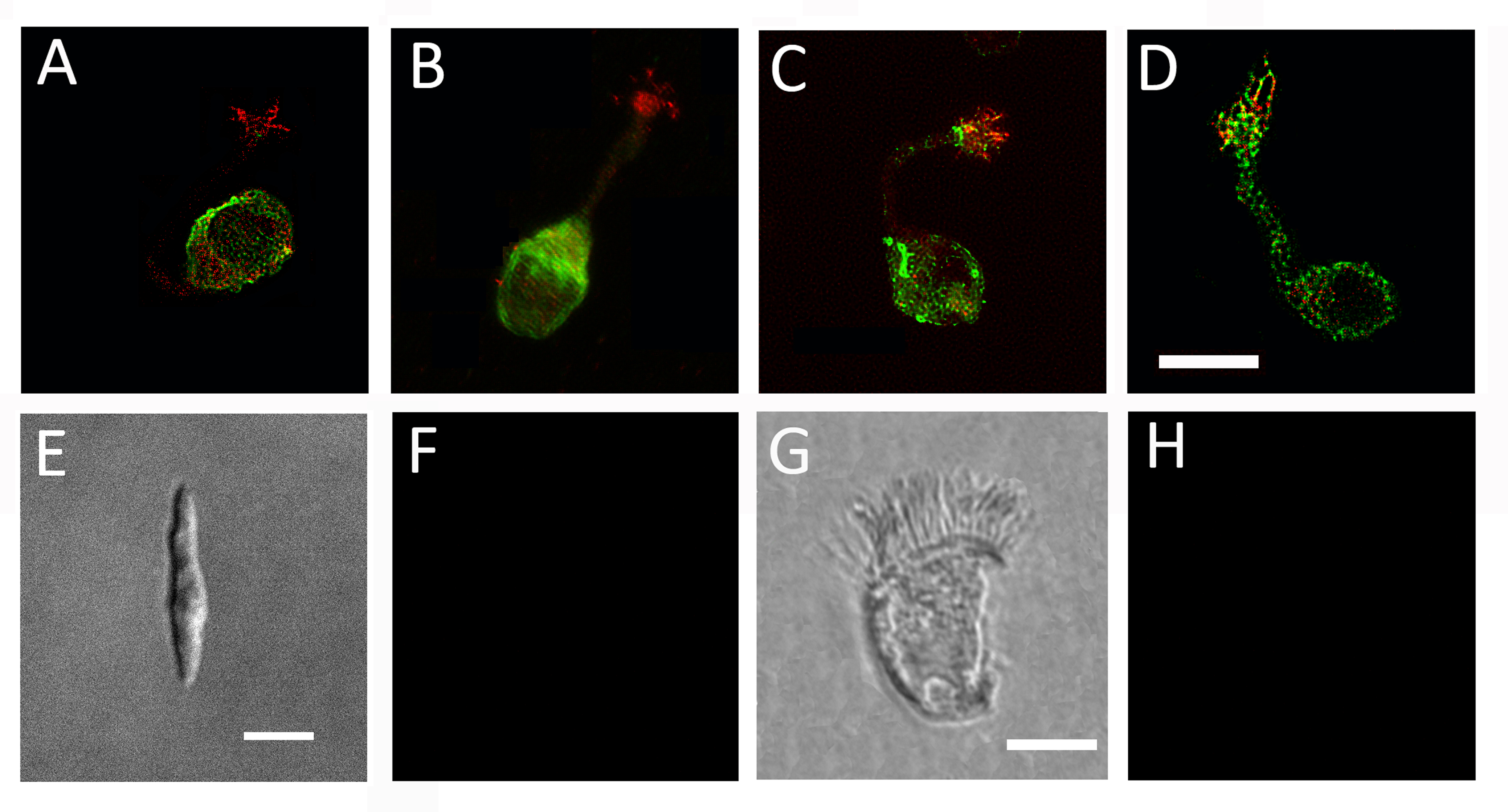

Supplement: Figure S3 — Four examples of immunolabeling of dissociated olfactory neurons (A–D) and lack of immunolabeling of cells with morphology different from OSNs (E–H). OSNs in A and B do not show immunolabeling to ClCa in the cilia while those in C and D do immunolabel for ciliary ClCa. A–E and G were co-labeled with anti-ClCa (green) and anti-CNGA2 (red) antibodies. E and G are DIC corresponding to F and H. Bars are 5 µm. (TIF) [file pone.0069295.s003.tif]

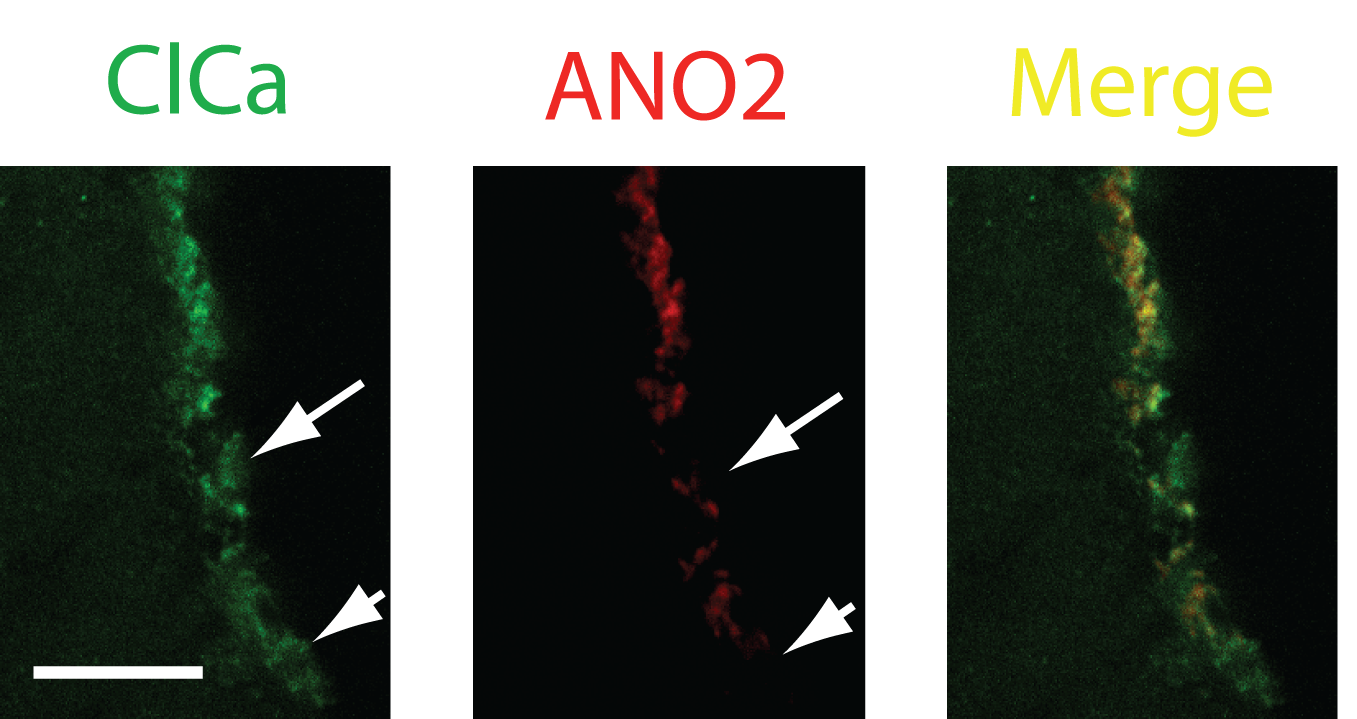

Supplement: Figure S4 — High magnification image of the ciliary layer of the olfactory epithelium with anti-ClCa (green), anti-ANO2 (red). While the cilia layer is labeled by both antibodies with significant overlap there are areas of the ciliary layer where these two antibodies do not overlap (arrows). Bar is 10 µm. This was obtained as a single image with a 60x oil objective. (TIF) [file pone.0069295.s004.tif]

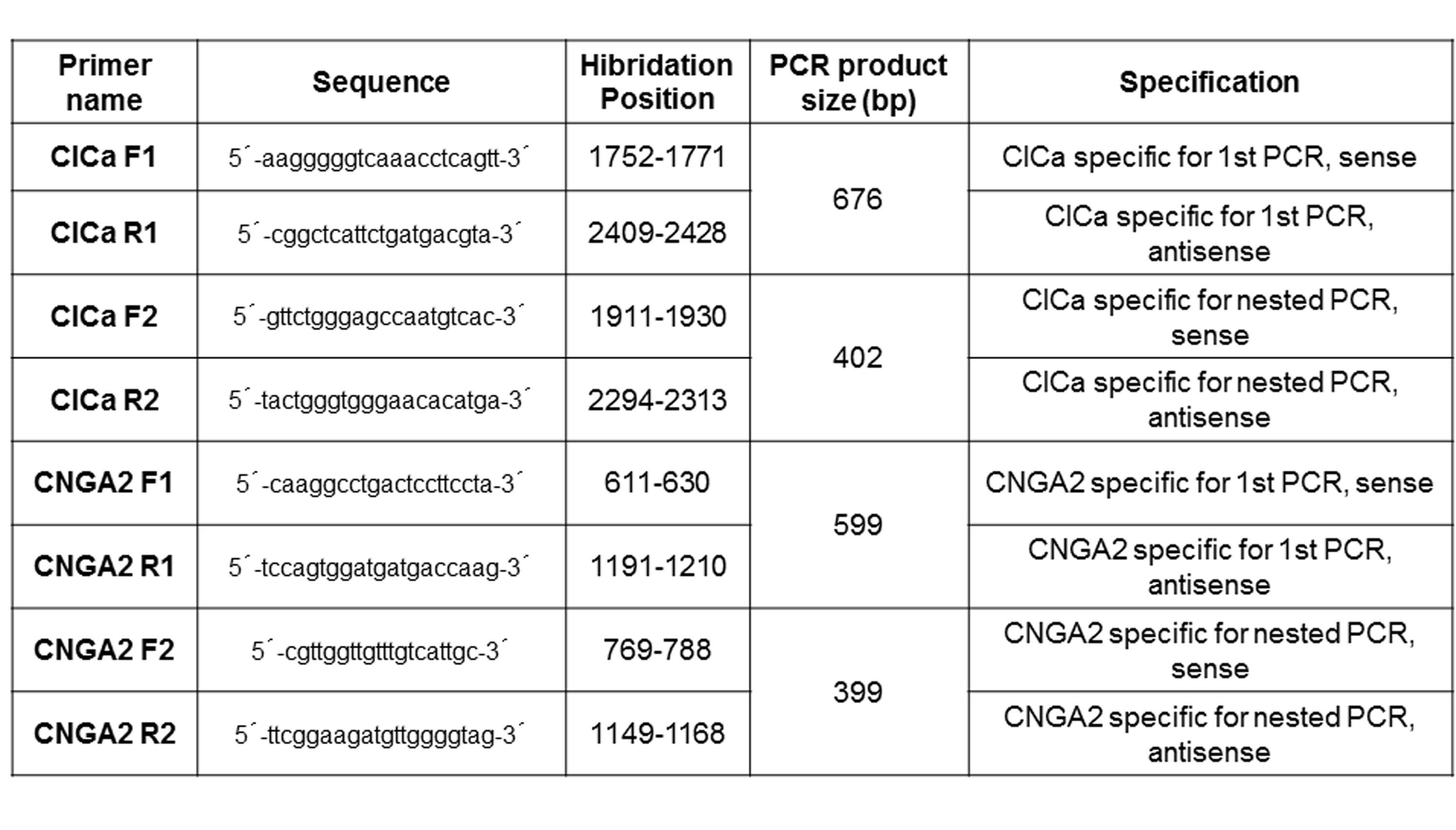

Supplement: Table S1 — Information for nested primers for ClCa and CNGA2 ( Fig. 1 ). The table includes the sequence and specification of each nested primer used to amplify mRNA of ClCa and CNGA2 to obtain the PCR products shown in Fig. 1. Forward (F) and reverse (R) primers for the first (F1/R1) and second (F2/R2) rounds of amplification are included. (JPG) [file pone.0069295.s005.jpg]

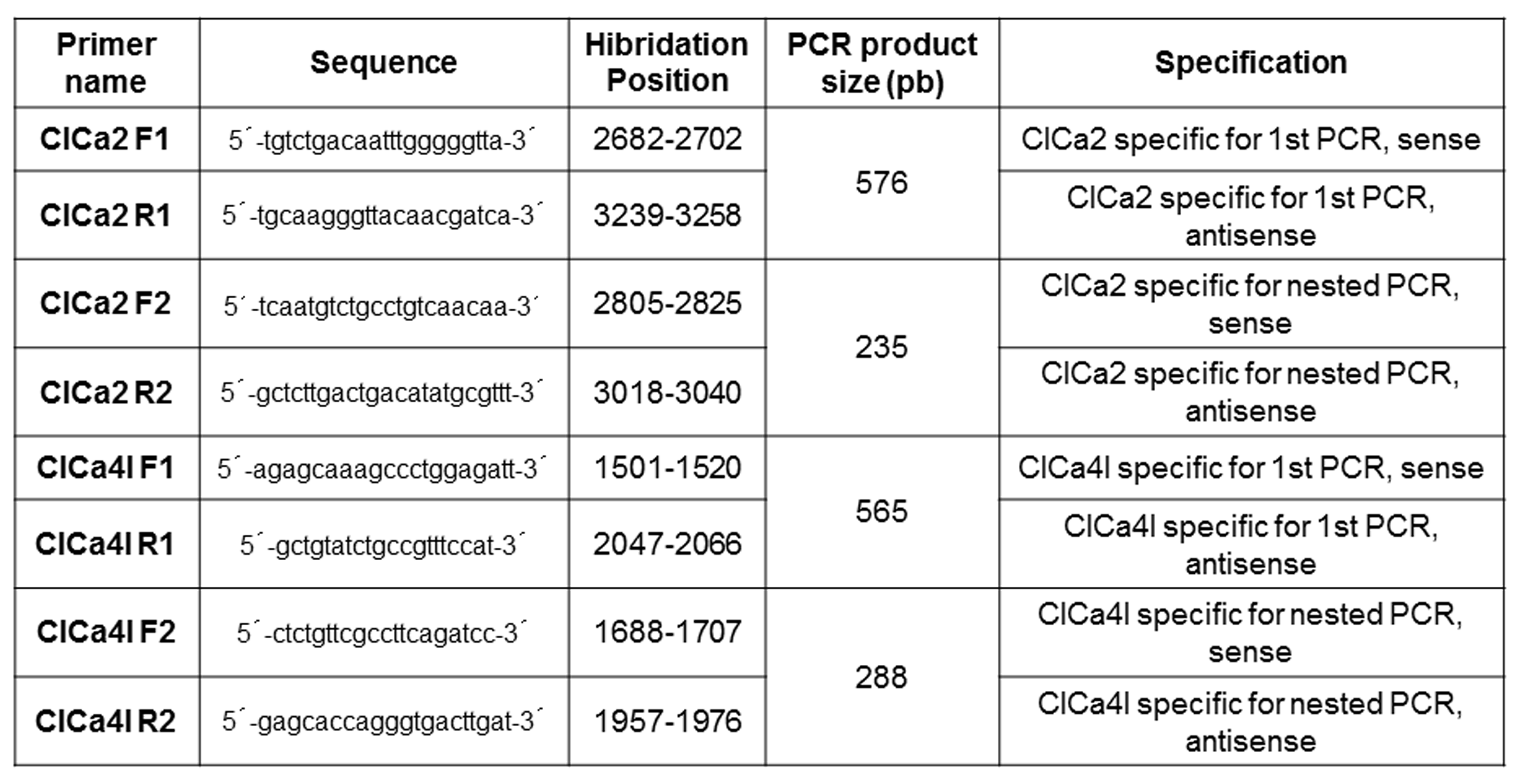

Supplement: Table S2 — Information on specific nested primers for ClCa2 and ClCa4l ( Fig. 2 ). The table shows the sequence of the specific ClCa2 and ClCa4l nested primers. (TIF) [file pone.0069295.s006.tif]

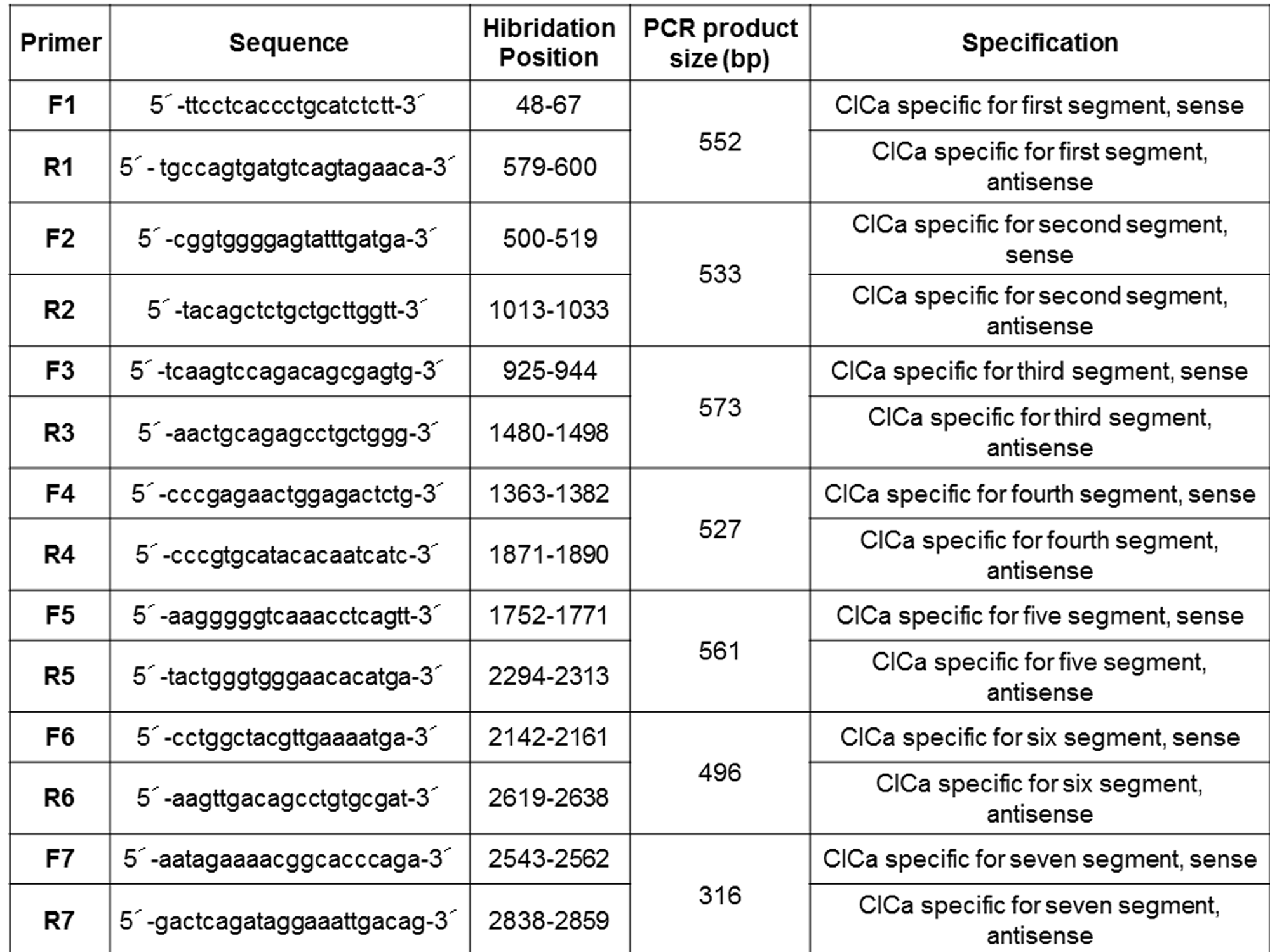

Supplement: Table S3 — Primer information for PCR used to determine the full sequence of the olfactory ClCa transcript. The table shows information on the seven pairs of primers used to amplify the entire sequence of the olfactory epithelium ClCa mRNA. The position refers to the sequence of ClCa4l (GeneID: 499721). These fragments and the alignment of the sequence with ClCa2 (GeneID: 362052) and ClCa4l are shown in a graphic form in Fig. 3. In addition, the sequences of each fragment with ClCa4l are shown in Fig. S1. Because of their high sequence similarity these primers would be able to amplify PCR products from both ClCa4l and ClCa2. (TIF) [file pone.0069295.s007.tif]

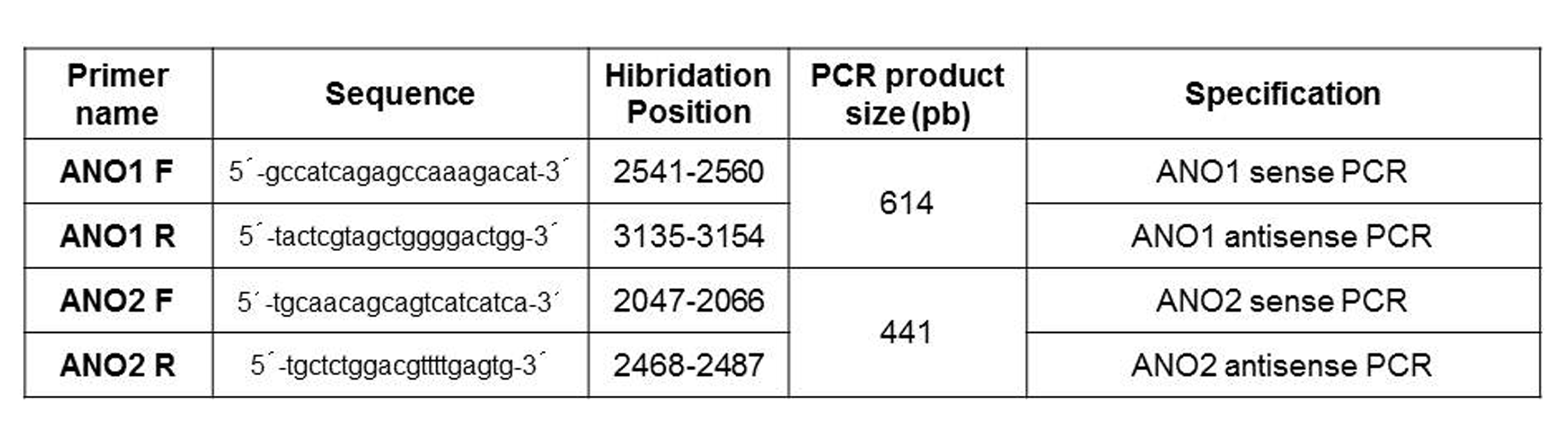

Supplement: Table S4 — Information on specific primers for ANO1 and ANO2 (Fig. S2). The table shows the sequence of the specific ANO1 (GeneID: 309135) and ANO2 (GeneID: 243634) primers. (TIF) [file pone.0069295.s008.tif]
